# Prevalence and risk factors of hepatitis B infection among mothers and children with hepatitis B infected mother in upper Dolpa, Nepal

**DOI:** 10.1186/s12879-017-2763-4

**Published:** 2017-10-10

**Authors:** Purusotam Raj Shedain, Madhu Dixit Devkota, Megha Raj Banjara, Huang Ling, Subash Dhital

**Affiliations:** 1 Department of Community Medicine and Public Health, Institute of Medicine/Ministry of Health, RamshahPath, Kathmandu, Nepal; 2 Department of Community Medicine and Public Health, Institute of Medicine, Tribhuvan University, Kathmandu, Nepal; 3 Central Department of Microbiology, Tribhuvan University (TU), Kirtipur, Kathmandu, Nepal; 4 Department of Preventive Medicine, Medical College of CTGU/Department of Community Medicine and Public Health, Institute of Medicine, Kathmandu, Nepal; 5 National Public Health Laboratory, Department of Health Services, Teku, Kathmandu, Nepal

**Keywords:** Children, Hepatitis B infection, Mothers, Nepal, Upper Dolpa

## Abstract

**Background:**

Hepatitis B Virus (HBV) infection is a worldwide public health problem. In Nepal, the prevalence of HBV is found to be low (0.9%), although high prevalence (≥8%) of HBV infection is depicted among subgroup/population in the mountain region by various studies. This study assessed the prevalence and the risk of HBV infection among mothers, as well as among the youngest child under 5 years old living with hepatitis B positive mothers in Dolpa, the most remote mountain district of Nepal.

**Methods:**

The cross sectional study survey was conducted between June and July 2014. All mothers with their youngest child under 5 years old were invited to participate in the survey and tested for hepatitis B surface antigen (HBsAg). The HBsAg positive mothers were further tested by 5-panel HBV test card. Children living with HBsAg positive mothers were also tested for HBsAg.

**Results:**

One hundred fifty-one mothers, comprising 37% of the total study population in the selected Village Development Committees (VDCs), were surveyed in the mobile health camps. The seroprevalence of HBsAg among mothers and their youngest child under 5 years old living with HBsAg positive mothers were 17% (95% CI, 11.01–22.99%) and 48% (95%CI, 28.42–67.58%) respectively.

The majority of HBV infected mothers were indigenous (84%) followed by Dalit (4%) and other castes (12%). Among HBV infected mothers, 40% were hepatitis B envelope antigen (HBeAg) positive. The prevalence of HBsAg was higher among children living with HBeAg positive mothers as compared to HBeAg negative (60% vs 40%) and male children compared to female (60% vs 33%). Thirty-six percent of children were vaccinated with a full course of the hepatitis B vaccine. Of these vaccinated children, 56% were HBsAg sero-positive.

**Conclusions:**

The HBV infection rate is high among mothers and children living with HBsAg positive mothers in the indigenous population of the most remote mountain community of Nepal.

## Background

Hepatitis B Virus (HBV) infection is a worldwide public health problem. Two hundred and forty million people are estimated to be chronically infected, particularly in low and middle income countries [1–3].HBV poses a serious concern worldwide as the virus is the major cause of chronic hepatitis, cirrhosis, and eventually hepatocellular carcinoma (HCC). The prevalence of HBV is highest in Sub-Saharan Africa and East Asia where 5–10% of the general population is infected, while in the Indian Subcontinent prevalence is estimated to be 2–5% [3]. Although most of the infections are self-limiting, the remainder persist as chronic infections in carrier individuals [2]. Around 15–40% of HBV infected patients develop cirrhosis, liver failure, or HCC [4]. Approximately 500,000 to 1.2 million people die of complications related to HBV infection annually [5].

In Nepal, the prevalence of HBV in the general population is believed to be heterogeneous, though there is no representative study or survey to confirm the national prevalence among the adults [6–8]. Most of the studies have depicted low prevalence of hepatitis B surface antigen (HBsAg) seroprevalence among the general population and blood donors, ranging from 0.4–1.2% [9–11].

A serosurvey of children born before and after hepatitis B vaccine introduction revealed that the prevalence of infection in pre-vaccination (10–12 years) and post-vaccination (5–6 years) cohorts of children were 0.28 and 0.13% respectively [12]. However, the prevalence of HBV infection is not homogenous due to differences observed within regions as well as ethnic groups. The Sherpas and Gurung residing at northern border of the country have a higher prevalence. HBsAg seroprevalence was found to be 7.35% of healthy individuals in Manang and 3.5% of individuals in Gurung at Mt. Everest Base camp [13]. Similarly, many other studies have suggested that there is high prevalence (≥8%) of hepatitis B infection among subgroups in mountain region of Nepal, ranging from 21 to 38% [8, 14–16].

In endemic areas, most infections occur in infants and children as a result of maternal-neonatal transmission or close childhood contact [17]. Children who are not HBV infected at birth remain at risk from long-term interpersonal contact with their infected mothers [18]. The disease outcome is determined by the time of exposure as early neonatal exposure has high morbidity and mortality rates [19, 20].

The determining factor for progression of acute HBV infection to the chronic lifelong carrier stage is the patient’s age at acquisition of the virus. Thus children acquiring infection perinatally or within 6 years of age are prone to develop chronic infection [21].

This study was conducted to determine the prevalence and identify the risk factors of hepatitis B infection among the children with a hepatitis B infected mother in upper part of Dolpa district, the most remote part of Nepal.

## Methods

### Study site

Dolpa is the most remote district of Nepal with 23 Village Development Committees (VDCs). Five VDCs (Chharka, Dho, Mukot, Saldhang and Tinje) of the upper part of the Dolpa were purposely selected for the study because these areas are home for the indigenous people that are highly mobile due to trade, seasonal migration, grazing cattle, and pilgrimage [14, 22].

### Study design and participants

A cross-sectional survey was performed among the target population of mothers with their youngest child under 5 years old. The sample size was based on a population of 503 children under 5 years old given by the Department of Health Services (DOHS, fiscal year 2014/2015), and the proportion of mothers with children under 5 years old (0.804) as per the Nepal Demographic Health Survey (NDHS 2011). The estimated total population was 404 individuals. Given the parameters, *N* = 404, Level (α) = 0.05, Tolerance (δ) = 0.05, and Prevalence (ρ) = 0.19 (from various studies), the required sample size would be 150 individuals with simple random sampling. A significant portion of *N* = 151, which comprised 37% of the total population, participated in this study.

All mothers with their youngest child under 5 years old were invited to participate in the survey. Written/verbal consent was obtained prior to the survey from the mothers for themselves and their children.

Two to three camps were organized in each VDC to ensure access and coverage of the survey in their cluster settlements. Participants were informed about the camps through Female Community Health Volunteers (FCHV), Amchi (local traditional doctors), local health workers, and non-governmental organization (NGO) staff working in the areas.

The participants were counseled on HBV infection including how to protect themselves from the infection, how to maintain health if they were infected, ways to protect their children from transmission of infection, and where to get vaccines if they were not immunized or not fully immunized.

### Sampling

Mothers with their youngest child under 5 years old and the youngest children of the HBsAg positive mothers who attended the camps were all enrolled in the study. The camps were organized in early June to late July 2014.

### Inclusion and exclusion criteria

Mothers with their youngest child under 5 years old were eligible for the study. Respondents who were not able to donate blood samples due to history of bleeding disorders, such as hemophilia, and unable to consent due to severe mental illness were not included in the survey.

### Data collection

#### Sociodemographic data collection

A structured questionnaire was administered to HBsAg seropositive mothers. Information on sociodemographic variables; history of antenatal care, delivery, and postnatal care services during the pregnancy of their youngest child; risk factors for hepatitis B infection; and vaccination history of mothers and children for hepatitis B virus were collected.

### Blood specimen collection

A 3.5 ml blood sample was collected from mothers and a 1 ml blood sample was collected from children by venipuncture. Samples were centrifuged and serum from both mothers and children were tested for HBsAg at the sites with SD Bioline(Standard Diagnostic, Inc., Korean, Sensitivity 100%, 95% CI 96.5–100%, Specificity 100%, 95% CI 97.9–100%).Serum sample was transported to the Research Laboratory, Institute of Medicine, maintaining cold chain. Serum of HBsAg positive mothers were further tested with a 5panel HBV test card (LumiQuick) with accuracy for HBsAg99.6%, HBsAb97.9%, HBcAb97.4%, HBeAg99.6%, and HBeAb98.2%. The 5panel HBV test card is an immunochromatographic test involving antibody or antigen sandwich and competitive immunoassay. All positive samples and random selection of 10% of negative samples were tested by enzyme-linked immunosorbentassay [23] for an HBsAg at the National Public Health Laboratory (NPHL), Teku Kathmandu.The sensitivity and specificity of the HBsAg rapid test was similar when compared with enzyme-linked immunosorbent assay.

### Data analysis

Data was entered in EpiData 3.1 and analyzed by SPSS 20 version. Descriptive analysis was performed.

## Results

### Description of the study population

**Fig. 1:**
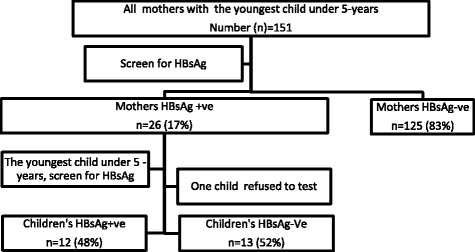
Research participants

One hundred fifty-one mothers attended the mobile health camp, which comprised 37% of the total study population. Among them, 125 mothers (83%) were HBsAg seronegative and the remaining 26 mothers (17%) were HBsAg seropositive. The proportion of HBsAg positive mothers was significantly lower than the proportion of HBsAg negative mothers in the population with the difference being greater than 2 standard error (SE).One child refused to give a blood sample, therefore only 25 mother-children pairs were included in the data analysis (Fig. 1).

### Infected mothers by sociodemographic characteristics, condom use, and antenatal care services

The majority of HBsAg positive mothers (52%) were from two VDCs, Chharka and Dho. The median age of mothers was 32 years, ranging from 19 to 46 years. The majority of the mothers belonged to Janajati group (84%) followed by Dalit (4%) and other groups (12%). Most of the mothers (96%) had never attended school (Table 1).

**Table 1:** The residence, age and caste/ethnicity of the HBsAg sero-positive mothers

Characteristics	Number (*N* = 25) and Percentage	
	n	%
Residence (VDCs)		
Chharka	5	20
Dho	8	32
Mukot	4	16
Saldang	4	16
Tinje	4	16
Age		
19–24 Years	2	8
25–29 Years	8	32
30–34 Years	7	28
35 Years and higher	8	32
Mean	32.04	
Range	19–46	
Cast/ethnicity		
Gurung/Lama (Indigenous)	21	84
Rokaya/Budha	3	12
BK	1	4

More than half (60%) of the participants had heard about the infection; however, only 16 % were tested for the hepatitis B infection in the past, and about 12% were informed about the status of their hepatitis B infection. An overwhelming number of participants (92%) had never had vaccination against hepatitis B infection.

None of the mothers used condoms during their last episode of sexual intercourse. Less than one-third (28%) of mothers had utilized antenatal care (ANC) services during the pregnancy of their youngest child. Some mothers (16%) had taken anthelminthic medication and nearly one-third (32%) had taken iron tablets or syrup during their most recent pregnancy (Table 2). Alcohol consumption was common among the participants (76%) with daily usage in a high proportion of the users (42%). The *Jand* and *Chhayang* (local fermented alcohols) were the most common (84%) form followed by homemade alcohol (16%). Only a few mothers (8%) consumed alcohol containing drinks less than once a week (Table 2).

**Table 2:** ANC services and alcohol consumption during the last pregnancy

ANC services and alcohol consumption	N	%
ANC services		
Yes	7	28
No	18	72
De-worming		
Yes	4	16
No	21	84
Iron tab or syrup		
Yes	8	32
No	17	68
Drinks containing alcohol during the last pregnancy		
Yes	19	76
No	6	24
Regularity of alcohol consumption during pregnancy (among users)		
Every day	8	42
2–3 times a week	8	42
At least once a week	1	5
Less than once a week	2	11
Form of alcohol taken during the pregnancy (among users)		
*Jand (raw* local fermented alcohol)	4	21
*Chhyang* (filter local fermented alcohol)	12	63
Homemade alcohol	3	16

### Characteristics of the children by sex, size at birth, and newborn care

Birth weight was obtained by recall from mothers. Less than half (40%) children were average size at birth, one-fifth were smaller than average, and one-third were larger than average. All children were born at home without skilled birth attendants and most (64%) were not fed colostrum (Table 3). Unsterile knives and scissors (96%) were commonly used instruments for cord cutting.

**Table 3:** Sex, size at birth and newborn care of the children

Children’s characteristics	Number	Percentage
Sex of baby		
Male	13	52.0
Female	12	48.0
Perceived Size of the child at birth		
Very large	2	8.0
Larger than average	7	28.0
Average	10	40.0
Smaller than average	5	20.0
Very small	1	4.0
Cord cutting instruments		
New/boiled blade	1	4.0
Knife/ Scissor	24	96.0
Colostrums feeding		
Yes	9	36.0
No	16	64.0

### Prevalence of hepatitis B infection

The seroprevalence of HBsAg among mothers and the youngest children living with HBsAg positive mothers was 17% (95% CI, 11.01–22.99%) and 48% (95% CI, 28.42–67.58%) respectively.

### Characteristics of mothers and status of their children’s HBsAg seroprevalence

The HBsAg seroprevalence was higher among children who lived with mothers having no ANC services (71.4%) compared to those having some ANC services (28.6%). The prevalence was higher among children with mothers that drank alcohol during pregnancy (57.9%) compared to those that did not (16.7%). The prevalence was higher among children with mothers that consumed alcohol daily (63%) (Table 4).

**Table 4:** Mother’s socio-demographic, serological characteristics and HBsAg sero-prevalence of children

Mother’s characteristics	Children’s HBsAg sero-prevalence, n (%)		*P* value*
	Yes	No	
Age			
Below 30 years	6 (50)	6 (50)	.848
30 and highest	6 (46.2)	7 (53.8)	
Caste/ethnicity			
Janajati	10 (47.6)	11 (52.4)	.930
Others	2 (50)	2 (50)	
ANC visit			
Yes	2 (28.6)	5 (71.4)	.225
No	10 (55.6)	8 (44.4)	
De-worming			
Yes	2 (40)	3 (60)	.689
No	10 (50)	10 (50)	
Iron taken			
Yes	3 (37.5)	5 (62.5)	.471
No	9 (52.9)	8 (47.1)	
Drinks containing alcohol during pregnancy			
Yes	11 (57.9)	8 (42.1)	.078
No	1 (16.7)	5 (83.3)	
Regularity of alcohol consumption during pregnancy (among users) (*n* = 19)			
Every day	5 (62.5)	3 (37.5)	.729
2–3 times to less than once week	6 (54.5)	5 (45.5)	
Form of alcohol taken during the pregnancy (among users %))			
Jand/Chhyang	8 (50)	8 (50)	.107
Homemade alcohol	3 (100)	–	
Mother’s HBeAg			
Positive	6 (60)	4 (40)	.327
Negative	6 (40)	9 (60)	
Mother’s HBeAb			
Positive	1 (11.1)	8 (88.9)	.002
Negative	11 (68.8)	5 (31.2)	

*Calculated using chi-square test

The prevalence of HBeAg and HBeAb seropositive mothers was 76% with 10 mothers (40%) HBeAg positive and 9 mothers (36%) HBeAb positive out of a total of 25 mothers. All mothers were HBcAb positive. The majority of children (60%) with HBeAg positive mothers were HBsAg positive, whereas less children (40%) were HBsAg positive with HBeAg negative mothers. Less children (11%) were HBsAg positive with HBeAb positive mothers, where as an overwhelming number of children (69%) were HBsAg positive with HBeAb negative mothers (Table 4).

### Characteristics of children and HBsAg seroprevalence

A greater proportion of male children (61.5%) were HBsAg seropositive compared to female children (33.3%). Children with average or greater size at birth were less frequently HBsAg seropositive (47.6%) compared to those that were smaller than average size at birth (50%). Children who were fed colostrum within 1 h after delivery were less HBsAg seropositive (44.4%) compared to no colostrum fed ones (50%) (Table 5).

**Table 5:** Children’s characteristics and HBsAg sero-prevalence

Children’s characteristics	HBsAg sero-prevalence, n (%)		*P* value*
	Yes	No	
Sex of baby			
Male	8 (61.5)	5 (38.5)	.159
Female	4 (33.3)	8 (66.7)	
Size of baby at birth			
Average and above	9 (47.4)	10 (52.6)	.910
Smaller than average	3 (50)	3 (50)	
Cord Cutting Instruments			
New/boiled blade	0 (0.0)	1 (100)	.327
Other (knife/sickle/khukuri/ scissor)	12 (50)	12 (50)	
Colostrums feeding			
Yes	4 (44.4)	5 (55.6)	.790
No	8 (50)	8 (50)	
Hepatitis B vaccination, n (%)			
Hep (1), 22 (88)	11 (50)	11 (50)	0.680
Hep (2), 17(68)	8 (47)	9 (53)	
Hep (3), 9 (36)	5 (56)	4 (44)	

*Calculated using chi-square test

**Fig. 2:**
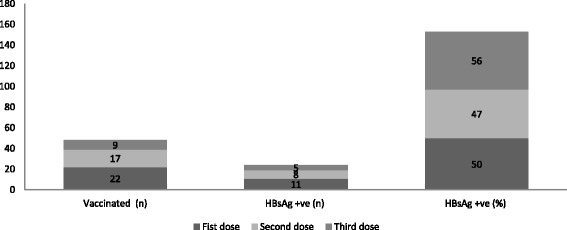
Children’s vaccination and HBsAg sero-prevalence

Administration of the first dose of the hepatitis B vaccine was high (88%) among the children; however, only half of the children that had been vaccinated were HBsAg seronegative. Nearly two-thirds of children (68%) were vaccinated with the second dose, but nearly half of them (53%) were HBsAg seronegative. Only one-third (36%) of children were vaccinated with full series of the hepatitis B vaccine (^HepB3^), although more than half of children (56%) were HBsAg seropositive (Table 5 and Fig. 2). Furthermore, the percentage of HBsAg seropositivity increased in older age groups with rates of 20, 50, and 72% respectively in age groups 0 to 12 months, 12 to 24 months, and 24 to 60 months (detailed data not shown).

## Discussion

The prevalence of HBsAg, if equal or greater than 8% is considered as high endemic [1]. This study showed that the prevalence of hepatitis B infection is very high among the mothers, and the youngest children under 5-years living with HBsAg sero-positive mothers in the upper part of the district, whereas the national prevalence of the infection in the general population is considered low (0.9%) [6]. Hence, it can be described as an issue of health disparity affecting indigenous mothers and children in Nepal.

Among HBV infected mothers, 40% were carried HBeAg seroprevalence. The majority of the children living with HBeAg positive mothers were HBsAg positive. Obviously, in highly endemic areas with HBeAg positive mothers, HBV is most commonly spread from mother-to-child at birth or perinatal transmission (WHO, #1292;Li, 2015 #1200;WHO, 2017 #1424). But in this study the proportion of positivity has increased with age group, so it can be assumed that there was other horizontal mode of transmission along with vertical one, which calls for a further study to know the relationship.

In our study 60% of children living with HBeAg positive mothers were found to be HBsAg positive, which was higher than sub-Sahara Africa (38.3%) between 3 and 12 months of age and lower than many other studies (70–90%) in Asia; whereas 40% of children living with HBeAg negative mothers were HBsAg positive, it is higher than sub-Saharan Africa (4.8%) [24] but in concordance with many other studies (< 10–40%), in the absence of post exposure immunoprophylaxis [25–27].

A significant proportion of children were HBsAg negative who were living with HBeAb positive mothers as in other studies [28]. It is due to the seroconversion from HBeAg to HBeAb, which usually indicates lower viral loads [29]. Further studies are required whether the seroconversion was due to spontaneous or local food or medicines as the “*Thikpa or Thigba*”, a local name of disease which is characterized by loss of appetite, distension of abdomen, yellowish discoloration of the conjunctiva/skin, dark urine are extensively treated by the local Amchi (a traditional doctor) and Lama with a various local herbs [8]. They perform wide ranges of health care activities such as chanting mantras for well-being and prescribing local herbal pills to cure the diseases as per their own diagnosis criteria and disease classification system [8, 14]. The Amchis also provide various surgical interventions to cure *Thigba/Thikpa* to their community through sucking, cutting and letting the blood from the big veins of the body but they are still unaware of proper sterilization practices and procedures [8], the impact of intervention needs to be assessed whether it has risked the transmission or prevented the infection in the community.

In this study male children were more infected than female ones. The proper explanations are not known, but male child is prone to most of infections compared to female child [30, 31]. Some of the studies had identified behavioral differences between sexes as an influencing factor, while other hypothesized that double X chromosome in females and random activation is also a factor for antibody development which may have protected the female child [32]. The explanation behind the cause of disparity of the prevalence of hepatitis B among sexes is not known. However, the disparity could have an impact on public health as it is estimated that the lifetime risk of death from Chronic Hepatitis B Infection (CHB) was 40–50% in men, and about 15% in women [33].

Using condom in their last sexual intercourse was nil. Utilization of ANC services was poor. It could be due to lack of awareness, unavailability of health care services and a far-flung settlement from the health post and district hospital. Only few (4%) mothers used a new/boiled blade to cut their newborns’ umbilical cord, majority of the mothers used old scissors and knives to cut the baby cord. There is a high chance of transmission of the infection while using the unsterilized contaminated scissors and knives. Furthermore, the poor availability of indicated items for infection prevention (0.2%) at health facilities in the mountain ecological region [34] could be a contributor to the high prevalence of the infection.

The National Immunization Program (NIP) has administered hepatitis B vaccine starting at 6 weeks of age [35] considering the low prevalence of the infection and other feasibility issues. Mothers were not screened for the HBsAg, an overwhelming numbers of participants had never had vaccinated against the HBV. Newborns were neither administered hepatitis B vaccine at birth nor hepatitis B immunoglobulin by the NIP. In the high endemicity setting (HBsAg prevalence ≥ 8%), the infection is mainly transmitted at birth or during early childhood (< 5 years), the first vaccine dose at birth are recommended, which prevent transmission of the infection by more than 90% of cases [36].

Only one-third of the children were administered three dose of hepatitis vaccine, among them more than half children were infected. The proportion of infection among the children increased with an increase in age groups. It has suggested that there is a horizontal transmission of hepatitis B infection among the children in addition to perinatal transmission. However, the perinatal and early neonatal transmission could be effectively prevented by at birth vaccination and immunoglobulin [2, 37]. Likewise, the horizontal transmission could be prevented with effective vaccination, awareness and education program. The finding has raised questions on the coverage of the NIP, the timing of the vaccination and effectiveness of the program.

Alcohol consumption was very common among the women and the infection prevalence was high among the children living with mothers who consumed drinks containing alcohol during their pregnancy compared to non drinkers. Generally, an alcohol use is associated with sexual risk behavior [38], though it requires a research for culture-specific and context-specific ways of dealing with the problems. There is a need for an in-depth understanding of the alcohol consumption that influences the risk behaviors for hepatitis B transmission and its consequences on them. Furthermore, both heavy alcohol consumption and hepatitis B are independent risk factors for the hepatocellular carcinoma [39, 40].

The majority of the infected mothers belonged to Janajati followed by Dalit and others. Janajati is Nepali word for indigenous people [41]. *Indigenous and Tribal Peoples Convention*, 1989 defines the indigenous people as the, tribal peoples in independent countries whose social, cultural and economic conditions distinguish them from other sections of the national community, and whose status is regulated wholly or partially by their own customs or traditions or by special laws or regulations.” In Nepali history the Janajatis have been excluded from the mainstream and these people are living in abject poverty [41]. Likewise, these people have been excluded from the modern health care services as they live in the remotest areas from the hospitals, have poor access to other promotive and preventive health care services [8].

### Limitations of the study

As far as we are aware, this is the first study ever done in the remotest hard-to-reach community of seminomadic population of upper Dolpa in Nepal. The geographically inaccessible setting; the silent nature of the infection; extremely mobile population; constraints of resources and the set objectives were the context.

The estimated target populations were 404. Given the parameters, *N* = 404, Level (α) = 0.05, Tolerance (δ) = 0.05 and Prevalence (ρ) = 0.19 (from a various studies), the required sample size would be 150 with the simple random sampling. A significant portion (*N* = 151, 37% of the total) of the population participated in this study. Camps were organized in each VDC to ensure access and coverage of the survey in their settlements. Participants were informed about the camps through FCHV, Amchi (local traditional doctors), local health workers, and non-governmental organization (NGO) staff working in the areas. We assume that there was no information bias as the target populations were informed through various social networks; there was no disease-bias as the chronic hepatitis B infection is often asymptomatic, even in advanced diseased stage; there was no selection bias as the all participants who attended the camps were enumerated; and researchers were bound to limit the methodology due to resources constraint.

Altogether, 151 participants attended the camps with their children, out of which (mothers) 26 were HBsAg positive and 125 were HBsAg negative. The prevalence of HBsAg in mother is 17%. The HBsAg positive prevalence proportion was significantly lower as compared to HBsAg negative mothers i.e. the difference between the proportion is greater than 2SE.

The sample size of interest of outcome (subset) is small (26 paired). One child refused to participate. Out of 25 children, 12 were HBsAg positive and 13 were HBsAg negative. Obviously the prevalence of HBsAg among children living with HBsAg positive mothers is higher than the WHO cutoff value (≥ 8). However, the difference between their proportions is not significant, i.e., < 2SE. On the basis, we can conclude that the prevalence of HBsAg among children living with HBsAg positive mothers is not significantly different from the HBsAg negative children living with HBsAg positive mothers. A further study with sufficient sample size is required to differentiate the discrepancy of the prevalence of the infection in the subset population.

## Conclusion

This study revealed that the prevalence of HBsAg is high among mothers and children living with hepatitis B positive mothers in the indigenous community of the upper Dolpa. In this endemic setting, the infection is mainly transmitted at birth or during early childhood. The hepatitis B vaccine series with the first dose at birth prevents the transmission of the infection in greater than 90% of cases.

A comprehensive intervention on prevention, control, and treatment are required in endemic areas to address the current and future associated liver health problems considering the high prevalence of the infection and additional risk factors, such as alcohol abuse, lack of safe sexual practices, poor access to modern health care services, and harmful traditional health care practices.

## Abbreviations

ANC: Antenatal care
CHB: Chronic hepatitis B infection
ELISA: Enzyme-linked immunosorbent assay
FCHV: Female Community Health Volunteers
HBcAb: Hepatitis B core antibody
HBeAb: Hepatitis B e antibody
HBeAg: Hepatitis B e antigen
HBsAg: Hepatitis B Surface Antigen
HBV: Hepatitis B Virus
HCC: Hepatocellular Carcinoma
IOM: Institute of Medicine
NGO: Non-Governmental Organization
NIP: National Immunization Program
NPHL: National Public Health Laboratory
TU: Tribhuvan University
VDC: Village Development Committees
WHO: World Health Organization
